# Precision Medicine in Graves’ Disease: CD40 Gene Variants Predict Clinical Response to an Anti-CD40 Monoclonal Antibody

**DOI:** 10.3389/fendo.2021.691781

**Published:** 2021-06-04

**Authors:** Larissa C. Faustino, George J. Kahaly, Lara Frommer, Erlinda Concepcion, Mihaela Stefan-Lifshitz, Yaron Tomer

**Affiliations:** ^1^Department of Medicine, Albert Einstein College of Medicine, New York, NY, United States; ^2^Department of Medicine I, Johannes Gutenberg University (JGU) Medical Center, Mainz, Germany

**Keywords:** gene, CD40, variant, Graves’ disease, precision medicine

## Abstract

**Background:**

CD40, a key co-stimulatory molecule expressed on antigen-presenting cells, is genetically associated with a number of autoimmune diseases including Graves’ disease (GD). Therefore, recent therapies targeting CD40 have been developed, including the anti-CD40 monoclonal antibody Iscalimab. In a recent pilot study, Iscalimab was shown to induce clinical remission in ~ 50% of GD patients, but the reason why only 50% of GD patients responded is not known. The aim of our study was to test the hypothesis that specific CD40 single nucleotide polymorphism (SNP) genotypes and haplotypes are associated with clinical response of GD patients to Iscalimab.

**Methods:**

We extracted genomic DNA from the whole blood of 13 GD patients treated with Iscalimab, and genotyped seven CD40 single nucleotide polymorphisms (SNPs) associated with autoimmunity. Additionally, we analyzed CD40 mRNA expression levels in whole blood. The patients’ CD40 SNP genotypes and mRNA levels were tested for association with clinical response to Iscalimab.

**Results:**

Three common haplotypes, designated haplotypes A, B, and C, were identified. Haplotypes B and C were associated with higher CD40 mRNA levels and clinical response to Iscalimab (i.e., patients achieving euthyroidism without need for additional medications), while haplotype A was associated with decreased CD40 mRNA levels and no response to Iscalimab.

**Conclusion:**

Our data suggest that genetic polymorphisms in the CD40 gene drive its expression levels and response to Iscalimab. Polymorphisms associated with higher CD40 levels are also associated with clinical response to CD40-targeted therapies. These results set the stage to implementing precision medicine in the therapeutic approach to GD.

## Introduction

Graves’ disease (GD) is one of the most common organ-specific autoimmune diseases affecting 1% to 2% of the adult population in the US (1, 2). GD is characterized by infiltration of the thyroid with autoreactive T-cells, and the production of thyrotropin receptor (TSHR) autoantibodies (Ab) that activate the TSHR and lead to the clinical manifestations of thyrotoxicosis and diffuse goiter (3, 4). Currently the treatment of GD is still based on three options: thionamides, radioactive iodine ablation of the gland, or surgical resection of the thyroid gland (5). All three therapeutic options for GD are aimed at suppressing the thyrotoxicosis but not at reversing the underlying autoimmune process. Therefore, patients are often difficult to manage and euthyroidism difficult to achieve (6–8). Even in patients that achieved biochemical euthyroidism with T4 replacement (after thyroid ablation) many still report symptoms and reduced quality of life (9–11). Therefore, there is a need for new curative therapies for GD targeting the autoimmune mechanisms underlying the disease. One such novel and exciting new therapy has been the development of a monoclonal antibody, Iscalimab (CFZ533), which targets the co-stimulatory molecule CD40 (12, 13).

Since CD40 has been shown to play a role in a number of autoimmune diseases including GD (14–17), Iscalimab has begun preliminary testing in several autoimmune conditions including myasthenia gravis (18), rheumatoid arthritis (19), Sjogren’s syndrome (20), and systemic lupus erythematosus (SLE) (21). Recently, Iscalimab (CFZ533) was reported to show potential effectiveness in a pilot study of 15 GD patients (22). All GD patients treated with Iscalimab showed significant reduction in TSHR-Ab, and 7 of 15 patients achieved biochemical euthyroidism (22). However, it is presently not known why only a subset of ~ 50% of patients responded. One potential explanation is that genetic variations at the CD40 gene-locus affect the clinical response to CD40-targeted therapies. We have previously identified a functional CD40 C/T single-nucleotide polymorphism (SNP) (rs1883832) that was associated with GD (14, 23). Following our discovery of the association of rs1883832 with GD, other CD40 SNPs were reported to be associated with autoimmunity [rs6074022 (24); rs745307, rs11569309, rs3765457 (25); and rs4810485 (15)]. Therefore, we hypothesized that one of these autoimmunity associated CD40 SNPs, or a haplotype consisting of a combination of them, may play a role in the clinical response of GD patients to Iscalimab.

In this study, we genotyped 13 GD patients that participated in the Iscalimab study and showed that specific CD40 SNP haplotypes were associated with clinical response of GD patients to Iscalimab.

## Materials and Methods

### Study Design

For full details of the study design see (22). The project was approved by the Johannes Gutenberg University (JGU) Medical Center Institutional Review Board and all patients provided signed informed consent. We analyzed DNA purified from patients that participated in the Iscalimab study. For a detailed description of the study protocol, inclusion and exclusion criteria, and outcomes see (22). Briefly, GD patients were enrolled at the endocrine clinic of the JGU Medical Center. GD was diagnosed by evidence of biochemical hyperthyroidism with thyrotoxic symptoms, and positive TSHR-Ab measured by the Roche Cobas Elecsys electrochemiluminescence immunoassay (Roche Diagnostic Corporation). The treatment period lasted 12 weeks and patients received 10 mg/kg Iscalimab IV at weeks 0, 2, 4, 8, and 12. Patients were followed up for 24 weeks after completing the Iscalimab treatment course. Worsening disease at follow-up was defined as relapse of symptoms and signs of thyrotoxicosis, or significant increase in serum thyroid hormone levels requiring additional medications. Response to Iscalimab was defined as normalization of thyroid functions at the completion of the study without the need for additional medications. Partial response was defined as normalization of peripheral free thyroid hormones with persistently suppressed serum TSH.

### DNA Purification and Genotyping

Genomic DNA was extracted and purified from whole blood using the QIAamp DNA blood kit (Qiagen, Hilden, Germany) following the manufacturer’s instructions. We genotyped six CD40 SNPs, previously shown to be associated with autoimmunity: rs6074022, rs1883832, rs745307, rs4810485, rs11569309, and rs3765457 (14, 15, 24–26), and another intergenic SNP, rs112809897, downstream the CD40 gene between the CD40 and CDH22 genes. These seven SNPs were genotyped using the Taqman allelic discrimination assays (Thermo-Fisher Scientific, Waltham, MA) as previously described (27). SNPs positions and locations are shown in **Table 1**.

**Table 1:** Distances and locations of the seven SNPs analyzed.

SNP	bp (hg38 Assembly)	Distance between SNPs	Location
rs6074022	46,111,557	0	Promoter
rs1883832	46,118,343	6,786	Kozak sequence
rs745307	46,118,447	104	intron 1
rs4810485	46,119,308	861	intron 1
rs11569309	46,119,911	603	intron 1
rs3765457	46,128,574	8,663	intron 8
rs112809897	46,149,941	21,367	Intergenic region between CD40 and CDH22

### Linkage Disequilibrium and Haplotype Analyses

Linkage disequilibrium (LD) and haplotype analyses were performed for SNPs rs6074022, rs1883832, rs745307, rs4810485, rs11569309, rs3765457, and rs112809897, all located at the chromosome 20q CD40 locus. For the LD and haplotype analyses we generated a pedigree (PED) file using the VCF to PED converter in the Ensembl database (https://useast.ensembl.org/index.html) for the CEU population. The generated PED file was then uploaded to the Haploview program which was used to perform the LD and haplotype analyses for the selected region containing these seven SNPs (28, 29). Haplotypes identified by Haploview were then analyzed and confirmed manually in our patients.

### Quantitation of CD40 RNA Levels Using RT-qPCR

Total RNA was purified from whole blood using QIAamp RNA blood kit (Qiagen) according to the manufacturer’s instructions. Total RNA was reverse transcribed using the Superscript III kit (Thermo Fisher Scientific) and RT-qPCR analyses were performed in a fluorescent temperature cycler (ABI PRISM 7300, Applied Biosystems). The sequences for the forward and reverse primers (Integrated DNA Technologies) were as follows: CD40: 5’-AAATGTCACCCTTGGACAAGCT-3’ and 5’-TTGTGCCTGCCTGTTGCA-3’; GAPDH: 5’-ATGGAAATCCCATCACCATCTT-3’ and 5’-CGCCCCACTTGATTTTGG-3’. After an initial incubation at 50°C for 2 min and 95°C for 10 min, the reactions were cycled 40 times using the following parameters: 95°C for 15s, 60°C for 30s, and 72°C for 45s. SYBR Green (Applied Biosystems) fluorescence was detected at the end of each cycle. The expression of CD40 was normalized to that of glyceraldehyde-3-phosphate dehydrogenase (GAPDH) by calculating the ΔCt of CD40 vs. GAPDH. We then calculated the ΔΔCt for each patient using the mean ΔCt for individuals carrying haplotype A as the reference expression levels. The fold change in CD40 mRNA expression levels (relative to the mean expression levels in individuals carrying haplotype A) was then determined using the 2^−ΔΔCt^ formula. All assays were repeated three times, and the averages of three experiments are shown. The results are expressed as fold change in CD40 mRNA levels relative to mean CD40 mRNA expression levels in individuals carrying haplotype A.

### Statistical Analyses

Statistical analyses were performed using the GraphPad Prism version 5.0 software. We used the Fisher exact test to test for association between CD40 SNP haplotypes and clinical response to Iscalimab treatment. The unpaired Student’s t-test was used to compare CD40 mRNA expression levels in GD patients carrying haplotype A vs. patients carrying either haplotype B or C. p < 0.05 was considered significant.

## Results

### Characteristics of Hyperthyroid GD Patients Treated With Iscalimab

Our cohort included 13 GD patients treated with Iscalimab (two patients from the original cohort (22) were not available for testing). GD was diagnosed as described above. **Table 2** shows the clinical, demographic, and serologic characteristics of the study patients, as well as their response to treatment with Iscalimab. The majority of patients were Caucasian women and the mean age was 49 years. Of the 13 patients included in our study 8 (61.5%) showed either response (7/13) or partial response (1/13) to Iscalimab as shown by decreased TSHR-Ab levels and/or normalizations of thyroid functions.

**Table 2:** Characteristics of the cohort of Graves’ disease (GD) patients analyzed in the study.

Patient	Age	Gender	Ethnicity	GD: New/Relapse	GO	Smoking	Baseline TSHR-Ab	Baseline TPO-Ab	Baseline Tg-Ab	Response to Iscalimab
1	40	F	Caucasian	New	Yes	No	151.5	915	125.1	Non-responder
2	40	F	Caucasian	Relapse	No	No	9.5	345	62.6	Responder
3	51	F	Caucasian	New	Yes	Yes	40.3	>1000	780.6	Responder
4	24	M	Caucasian	Relapse	Yes	Yes	171.3	193	1.3	Responder
5	19	F	Caucasian	New	No	Yes	11.4	122	3.4	Responder
6	49	F	Caucasian	Relapse	Yes	Yes	33.3	>1000	4	Non-responder
7	26	F	Caucasian	New	No	No	56.8	876	143	Responder
8	49	F	Caucasian	Relapse	No	No	6.7	977	6.3	Non-responder
9	50	M	Caucasian	Relapse	Yes	Yes	48.3	>1000	95	Partial response
10	51	F	Caucasian	Relapse	Yes	Yes	4.17	403	67	Responder
11	52	F	Caucasian	Relapse	No	No	11.0	173	3.9	Non-responder
12	65	F	Caucasian	Relapse	No	Yes	27.46	181	40.4	Responder
13	53	F	Caucasian	Relapse	No	Yes	2.54	61	1	Non-responder

Units and normal values of thyroid antibodies: TSHR-Ab, IU/L <1.75; TPO-Ab, IU/ml <25; Tg-Ab, IU/ml <40.

### Linkage Disequilibrium and Haplotype Analyses

**Figure 1:**
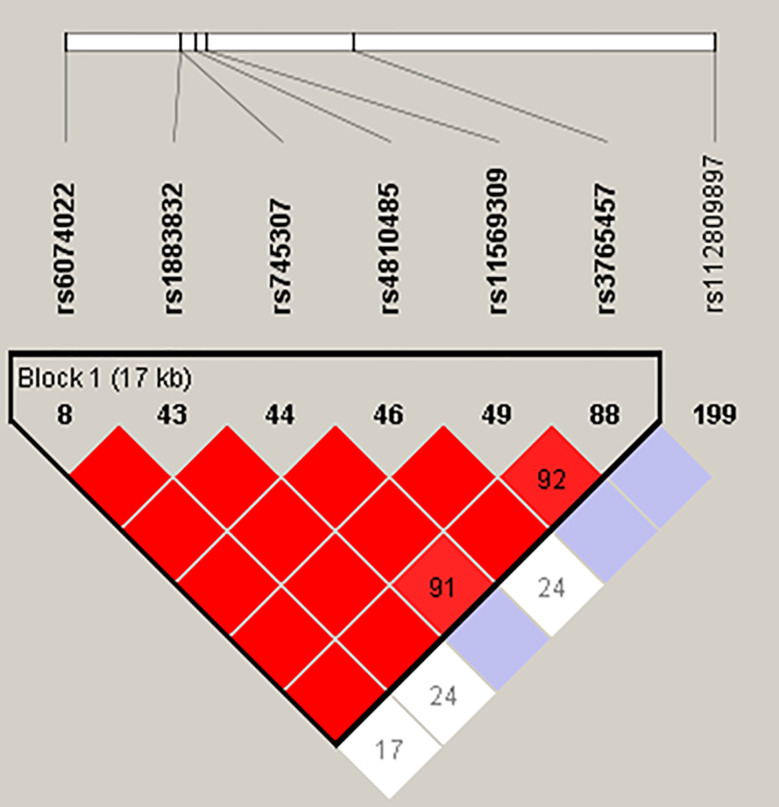
Haploview map of the seven CD40 SNPs analyzed in this study. The color scheme of the LD map ranges from white to red, with red indicating tight LD with a D’ of 1.

Linkage disequilibrium (LD) analysis, performed using Haploview, revealed tight LD between the six SNPs located in the promoter, 5’UTR, and introns of CD40 (rs6074022, rs1883832, rs745307, rs4810485, rs11569309, rs3765457) due to the close distances between them (**Figure 1**). Therefore, our haplotype analysis identified haplotypes for these six SNPs only. Haplotype analysis was performed using the Haploview program (28, 29) for the CEU population (see *Methods*). Haplotypes identified by Haploview in the CEU population were verified in our patients by manual analysis. We identified eight distinct haplotypes that we designated as A–H (**Table 3**). Of them, five were identified in both the CEU population and our patients (A-E; **Table 3**), one was identified only in the CEU population (F; **Table 3**) and two were identified only in our patients (G, H; **Table 3**). Of these eight haplotypes we identified three dominant haplotypes in our patients that were strongly associated with clinical response to Iscalimab (see below): Haplotype **A** (CTGTTAC); haplotype **B** (TCGGTAC); and haplotype **C** (TCAGTAC) (**Table 3**).

**Table 3:** Major haplotypes identified in the CEU population using Haploview, and in our cohort using manual analysis.

**Haplotype identified in:**	**Haplotype Name**	Haplotype Allelic Sequence*	**Frequency in the CEU population**
CEU (by Haploview) and Current cohort (manually)	**A**	**CT**G**T**TA	0.227
CEU (by Haploview) and Current cohort (manually)	**B**	**TC**G**G**TA	0.434
CEU (by Haploview) and Current cohort (manually)	**C**	**TC**A**G**TA	0.071
CEU (by Haploview) and Current cohort (manually)	**D**	TCAGCA	0.061
CEU (by Haploview) and Current cohort (manually)	**E**	TCGGTG	0.011
CEU only (by Haploview)	**F**	TCAGCG	0.191
			
Current Cohort only	**G**	CCGGTA	NA
Current Cohort only	**H**	CCAGCG	NA

SNPs included in the haplotypes in order (upstream to downstream CD40): rs6074022, rs1883832, rs745307, rs4810485, rs11569309, and rs3765457.

*The three key alleles in haplotypes A, B, and C that are associated with response or no response to Iscalimab are highlighted (see text).

NA, not applicable.

### Association Between CD40 SNP Haplotypes and Clinical Response to Iscalimab

Of the 13 patients in our cohort, eight responded to Iscalimab and five did not respond (**Table 2**). As shown in **Table 4** all eight patients that had a clinical response to Iscalimab carried either haplotypes B (**TC**G**G**TAC) or C (**TC**A**G**TAC) and all five non-responders carried haplotype A (**CT**G**T**TAC) [p=0.0008, using Fisher exact test]. Further analysis revealed three key SNPs differentiating responders from non-responders: rs6074022 (T allele associated with response to Iscalimab and C allele with no response), rs1883832 (C allele associated with response to Iscalimab and T allele with no response), and rs4810485 (G allele associated with response to Iscalimab and T allele with no response). Thus, using these three SNPs, haplotype TCG was associated with response to Iscalimab and haplotype CTT was associated with no response to Iscalimab (**Table 4**).

**Table 4:** CD40 SNP haplotypes and mRNA expression levels in 13 GD patients treated with Iscalimab.

Patient	Haplotype	Key haplotype sequence*	CD40 mRNA expression**	Response to Iscalimab
**1**	**A**/G	**CTT**	0.932	**Non-responder**
**2**	B	**TCG**	2.889	Responder
**3**	B	**TCG**	1.831	Responder
**4**	C	**TCG**	4.170	Responder
**5**	C	**TCG**	1.667	Responder
**6**	**A**/G	**CTT**	0.893	**Non-responder**
**7**	B	**TCG**	1.613	Responder
**8**	**A/**G	**CTT**	0.939	**Non-responder**
**9**	B	**TCG**	2.126	Partial responder
**10**	C/D	**TCG**	10.863	Responder
**11**	A***/H	**CTT**	1.217	**Non-responder**
**12**	B/E	**TCG**	11.560	Responder
**13**	**A**/G	**CTT**	1.051	**Non-responder**

*Key haplotypes were derived from SNPs rs6074022, rs1883832, and rs4810485 (in that order 5’ to 3’).

**CD40 mRNA expression levels were calculated by the – (ΔΔ Ct) method using the mean Δ Ct of non-responders as a baseline, and are expressed as the fold change relative to mean levels in non-responders (see Methods).

***In patient #11 haplotype A contained a sequence change from CT**G**TTA to CT**A**TTA, however the three key alleles in haplotype A (CTnT) are preserved (see **Table 3**).

### Association Between Response to Iscalimab and CD40 mRNA Expression Levels

We tested whether response to Iscalimab was also associated with CD40 mRNA expression levels in whole blood. mRNA levels of CD40 were significantly higher in responders to Iscalimab compared to non-responders (p=0.046; **Table 4**). Since all responders also carried haplotypes B or C and all non-responders carried haplotype A, this suggested that haplotypes B and C may drive higher expression levels of CD40, leading to clinical response to Iscalimab while haplotype A may drive lower expression of CD40 and reduced response to Iscalimab.

## Discussion

Precision medicine is a new field of medicine aimed at tailoring therapies to individual patients based on both the molecular mechanisms triggering their disease, as well as the patients’ unique genetic variants (30). Precision medicine has the potential to revolutionize medical care as tailoring treatments to individual patients will maximize the efficacy of medications while minimizing their side effects (31). In recent years, there have been many exciting developments in precision medicine approaches for autoimmune diseases (32, 33). Our study demonstrated, for the first time, that precision medicine could potentially be applied to the treatment of GD. We have shown in a pilot cohort of GD patients an association between specific CD40 SNP haplotypes, as well as CD40 mRNA expression levels and clinical response to CD40 targeted therapy with Iscalimab (**Table 4**). Therefore, if our data are confirmed in larger cohorts, it will enable precision tailoring of CD40 targeted therapies to individual patients carrying the B or C haplotypes and/or having higher CD40 mRNA levels in their blood (**Table 4**).

Genetic susceptibility has long been recognized to play a pivotal role in the etiology of GD (34). The CD40 gene, expressed on antigen-presenting cells, such as dendritic cells and B-cells (35), was shown to be a key susceptibility gene for GD (14), as well as other autoimmune diseases (15–17). We have previously mapped a functional SNP in the CD40 Kozak-sequence, rs1883832, that was associated with GD (14). Furthermore, we have shown that the GD risk allele (C) increased the expression of CD40 (23, 36), and that increased expression of CD40 on thyroid cells was associated with targeting the autoimmune response to the thyroid in GD (37, 38). The association of CD40 with GD has now been confirmed in many studies in different ethnic and geographic populations (39–45). Moreover, some studies have shown that CD40 polymorphisms can predict remission and relapse of GD (25, 44).

In view of the pivotal role of CD40 in the etiology of GD the newly developed monoclonal antibody, Iscalimab, which blocks CD40 activation, was tested in a cohort of GD patients, and was shown be effective in reducing TSHR-Ab in all patients and normalizing thyroid hormone levels in ~ 50% of hyperthyroid GD patients (22). Our data showed that haplotypes B and C were significantly associated with response to therapy while all carriers of haplotype A were non-responders. Moreover, we found that individuals carrying haplotype A had lower CD40 mRNA expression in their blood than patients carrying haplotypes B and C. This finding could provide a mechanistic explanation for the lack of response to Iscalimab therapy in GD patients carrying haplotype A. Since patients carrying haplotypes B and C had significantly higher levels of expression of CD40, it is likely that genetically-driven CD40 over-expression played a major role in the development of GD in them (36, 37), and therefore blocking CD40 was an effective intervention in them. On the other hand, GD patients carrying haplotype A had very low levels of CD40 expression and therefore, CD40 probably did not play an important role in the etiology of GD in them and other mechanisms likely contributed.

Intriguingly, all three SNPs comprising haplotypes A, B, and C were previously shown to influence CD40 gene expression levels with the GD risk allele of each SNP consistently associated with significantly higher CD40 expression levels. SNP rs6074022, located in the promoter of CD40, has been shown to be associated with CD40 mRNA expression levels in whole blood from patients with multiple sclerosis (MS) and healthy controls. MS patients and controls carrying the TT (AA) genotype had significantly higher levels of CD40 mRNA compared to patients carrying the CT (GA) and CC (GG) genotypes (46). Consistent with these findings, in our study, the T allele of rs6074022 was associated with higher CD40 mRNA expression in whole blood compared to the C allele (**Table 4**). SNP rs1883832, located in the Kozak sequence of CD40, was shown by our group to be associated with CD40 expression, with the CC genotype associated with significantly higher protein expression levels of CD40 compared to the CT and TT genotypes (23, 36). These results were replicated in this study showing that the C allele of rs1883832 was associated with significantly higher levels of CD40 than the T allele (**Table 4**). Finally, SNP rs4810485, located in intron 1 of CD40, was also shown to be associated with CD40 mRNA and protein expression levels in PBMCs from patients with SLE and controls. The GG genotype was associated with significantly higher CD40 mRNA levels in PBMC’s and significantly increased CD40 protein levels in peripheral blood CD14+ monocytes and CD19+ B cells compared to the GT and TT genotypes (16). Our findings are consistent with these data. We showed that the G allele of rs4810485 was associated with significantly higher CD40 levels compared with the T allele. However, since these three SNPs are in tight LD (**Figure 1**) it is not possible to determine which one is the causative SNP driving higher CD40 expression levels and clinical response to Iscalimab. Further studies are needed to determine which of these three SNPs is causative.

In conclusion, this was a pilot study on a small cohort of 13 GD patients. If our results are confirmed in larger cohorts, they set the stage to implementing precision medicine in the treatment of GD and potentially other autoimmune diseases. Genotyping of CD40 SNP variants could be utilized in pre-treatment screening potentially allowing clinicians to individualize treatment regimens based on a patient’s SNP genotype.

## Data Availability Statement

The original contributions presented in the study are included in the article/**Supplementary Material**. Further inquiries can be directed to the corresponding author.

## Ethics Statement

The studies involving human participants were reviewed and approved by the Johannes Gutenberg University (JGU) Medical Center Institutional Review Board. The patients/participants provided their written informed consent to participate in this study.

## Author Contributions

LFa: Performed experiments, data analysis and interpretation, and participated in writing the manuscript. GK: Data collection, data analysis, and participated in writing the manuscript. LFr: Data collection and participated in writing the manuscript. EC: Performed experiments. MS-L: Data interpretation and participated in writing the manuscript. YT: Conceptualization, literature search, data analysis and interpretation, and wrote the manuscript. All authors contributed to the article and approved the submitted version.

## Funding

This work was supported in part by grants DK067555 & DK073681 from NIDDK (to YT). The JGU Medical Center, Mainz, Germany has received research-associated funding from Novartis, Cambridge, MA, USA to perform a phase II trial of Iscalimab in patients with Graves’ disease (Clinical Trials Registration: NCT02713256). Novartis USA had no role in the present study design, data collection and analysis, decision to publish, or preparation of this manuscript.

## Conflict of Interest

YT, GK, and LFa declare that they submitted a Patent Application (US Provisional Application No: 63/120,514) entitled: “Method for predicting patient response to CD40-targeted therapies” which is related to the current manuscript. YT declares that he also submitted two additional patent disclosures that are not related to the content of this manuscript. YT was previously (January 2015 to June 2017) the PI on a basic research project jointly funded by the Juvenile Diabetes Research Foundation and Pfizer. The current manuscript is not related to that research project. GK consults for Immunovant, USA, Mediomics, USA, Merck, Germany, Novartis, Switzerland, and Quidel, USA.

The remaining authors declare that the research was conducted in the absence of any commercial or financial relationships that could be construed as a potential conflict of interest.
