# Supplementary material for: Precision Medicine in Graves’ Disease: CD40 Gene Variants Predict Clinical Response to an Anti-CD40 Monoclonal Antibody
Source: Front Endocrinol (Lausanne). 2021 Jun 4;12:691781. doi: 10.3389/fendo.2021.691781 (PMC8212124; doi:10.3389/fendo.2021.691781)
Supplement: Supplementary file 1 [file DataSheet_1.pdf]

Supplementary Table 1: Genotypes and haplotypes for 6 SNPs in tight LD located at the CD40 locus

Patient	rs6074022	rs1883832	rs745307	rs4810485	rs11569309	rs3765457	Haplotype Alleles	Haplotype Name	Response to Iscalimab
**1**	CC	TC	GG	TG	TT	AA	CTGTTA / CCGGTA	A / G	**Non Responder**
**2**	TT	CC	GG	GG	TT	AA	TCGGTA*	B / B*	Responder
**3**	TT	CC	GG	GG	TT	AA	TCGGTA	B / B	Responder
**4**	TT	CC	AA	GG	TT	AA	TCAGTA	C / C	Responder
**5**	TT	CC	AA	GG	TT	AA	TCAGTA	C / C	Responder
**6**	CC	TC	GG	TG	TT	AA	CTGTTA / CCGGTA	A / G	**Non Responder**
**7**	TT	CC	GG	GG	TT	AA	TCGGTA	B / B	Responder
**8**	CC	TC	GG	TG	TT	AA	CTGTTA / CCGGTA	A / G	**Non Responder**
**9**	TT	CC	GG	GG	TT	AA	TCGGTA	B / B	Partial Responder
**10**	TT	CC	AA	GG	TC	AA	TCAGTA / TCAGCA	C / D	Responder
**11**	CC	TC	AA	TG	TC	AG	CT**A**TTA / CCAGCG	A** / H	**Non Responder**
**12**	TT	CC	GG	GG	TT	AG	TCGGTA / TCGGTG	B / E	Responder
**13**	CC	TC	GG	TG	TT	AA	CTGTTA / CCGGTA	A / G	**Non Responder**

*In homozygote individuals we showed only one allele since both alleles are identical

In patient #11 haplotype A contained a sequence change from CTG**TTA to CT**A**TTA, however the 3 key alleles in haplotype A (CTxT) are preserved (see **Table 3**)
